# The Worker's Leisure

**Published:** 1921-03-12

**Authors:** 


THE PUBLIC WELL-BEING—(*continued*).

### The Worker's Leisure.

### A MODERN PROBLEM.

Contrasted with the war period, to-day we are a nation of "slackers." Then, working hours were synonymous with waking hours. Now, the acknowledged maximum is eight hours' work a day, and it is not too much to say that many people are definitely less happy for the change. That is no criticism of a reform that is almost in the order of Nature and was certainly long demanded even before the war, and generally regarded as both wise and just. As man progresses he expects to gain more command over the physical world, and from the time when little children worked sixteen hours or more a day we have advanced without a backward step to the long demanded eight-hour day.

The effect on industry has yet to be ascertained. At any rate, the extreme prophecies seem to have been belied. Industry has not been ruined, but, on the other hand, output has not, as some maintained that it would, kept up to the former level. The truth is that the experiment has not yet had time to settle down to the normal. This is also true of the effects on the other part of the worker's day, his leisure hours.

It is astonishing how little real attention social students and medical men have given to a change which may prove to have been the most revolutionary of our time. Many of our fathers would have thought it perfectly dangerous to allow the workers so much time to contract idle habits and to become restless. We have plunged into the great experiment, and its effects, which are already beginning to be apparent, and will within a few years be more important than all the Acts of Parliament we care so much about, are receiving only

**THE PUBLIC WELL-BEING**—*(continued)*.

casual attention, instead of being the subject of critical scientific study.

The merely physical effects will probably take half a generation really to become apparent, for their influence will not be fully felt by men who have worked the old hours during all the formative part of their life. But the mental effects will be more rapid, and may be more serious. Rome allayed the discontent of its citizens with circuses. Modern entertainments to a large extent are no more than organised drugs for the mind, alternately stimulating and stupefying, but providing no source of energy or mental driving-power. They allay boredom for a time, only to create eventually a chronic condition of boredom. And boredom may eventually become something as serious as a disease. Many of the best Labour leaders—men like Mr. J. R. Clynes—realise that the question of the worker's leisure may become as important as questions that concern his labour. Many large firms have begun to recognise the same thing, and, through their welfare schemes, are trying to stimulate their workers, especially the younger men and women, to take an interest not merely in studies, but in sport, games, and dancing. A conference was held in Manchester not long ago to consider no other question but this of the working man's leisure. It was attended by representatives of societies so various as to show a surprising amount of enterprise in catering for the worker's leisure hours. But, after all its discussions, it got no nearer to what is the real core of the problem.

The root trouble is to get the worker's interest in your schemes, and then to keep it. The modern worker has thousands of interests to which his grandfather was an entire stranger, but he moves from one to the other like a child at a party faced with so many dishes that he cannot enjoy a single one. There is here a condition in which the experience of the medical profession ought to be turned to good use. Every medical man knows how a single compelling interest changes the whole mental and physical tone of the patient. The mother becomes strong at the touch of the baby. The possibility of getting back to work helps a man past the dangerous corner, and a hold on some single theme of life makes a man a better patient and gives him a better chance of recovery. This is exactly what the worker wants in his leisure—something to unify his aims and his interests. Leisure spent in flitting about from the music-hall to the sports club and from that to the billiard-table, and then to occasional lectures, is better than mere idleness, but it needs a purpose. What is wanted is the something that makes all the difference between an education and years of desultory reading. Things which, thrown casually together, may be of little value, if they are grouped in a scheme of things become of real importance. It matters little what the overruling interest is, be it politics, social study, music, or education in its thousand forms; but every doctor can say from his own experience that the leisure of every worker needs to be informed by some guiding purpose, or at least some main interest, if the worker and the community for which his work is done are to reap a real advantage from his respite from the daily round.

